# Supplementary material for: Interfacial Enhancement by CNTs Grafting towards High-Performance Mechanical Properties of Carbon Fiber-Reinforced Epoxy Composites
Source: Materials (Basel). 2023 May 18;16(10):3825. doi: 10.3390/ma16103825 (PMC10224247; doi:10.3390/ma16103825)
Supplement: Supplementary file 1 [file materials-16-03825-s001.zip › materials-2360133-supplementary.pdf]

*materials*

MDPI

*Supplementary Materials*

# Interfacial Enhancement by CNTs Grafting towards High-Performance Mechanical Properties of Carbon Fiber-Reinforced Epoxy Composites

The surface morphology of the CNTs modified with KH560 was characterized by TEM and FTIR, as shown in Figure S1. The results demonstrate the successful grafting of KH560 onto the surface of CNTs (Figure S1-d, e, f). One coating layer of KH560 on the CNTs can be observed (Figure S1-a, b, c, f).

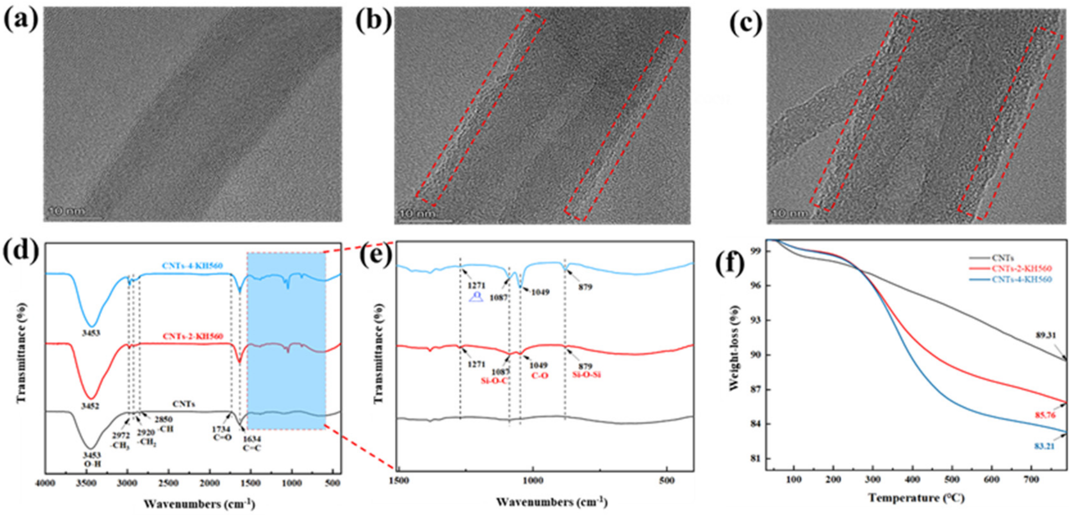


**Figure S1.** Morphology, chemical and thermal characteristics of CNTs before and after the modification of KH560.TEM images of CNTs: **(a)** pristine CNTs, **(b)** 2% KH560-modified CNTs (CNTs-2-KH560), **(c)** 4% KH560-modified CNTs (CNTs-4-KH560). FTIR spectra **(d)**, **(e)** and thermogravimetric analysis curves **(f)** of those three CNTs samples.

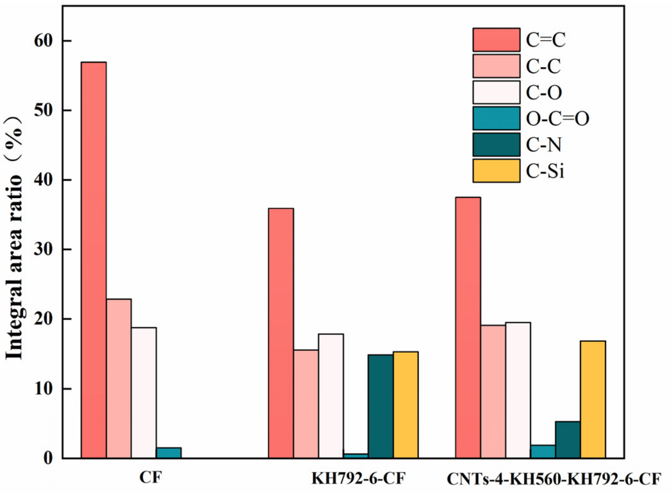

**Figure S2.** Comparison of the surface chemical composition of CFs before and after the modification of KH792 (KH792-6-CF) and loading of KH560-modified CNTs (CNTs-4-KH560-KH792-6-CFs), according to the deconvolution of XPS results shown in Figure 6.

## Experimental

The mechanical properties of the composites were tested using a material universal testing machine (UTM4304X, Shenzhen New Sansi Materials Testing Co.Ltd.). Referring to GB/T9341-2008, the bending strength test of carbon fiber/epoxy resin composite was carried out under normal room temperature and atmospheric pressure. The loading speed of external load was 2 mm/min, the span of specimen pieces was 50 mm and the average value was calculated for each group of 5 samples. The test results are shown in Figure S3.

The bending test results (Figure S3) showed that the bending strength of the prepared carbon nanotube-grafted modified carbon fiber-reinforced composites (1719 MPa) was increased by 33.7% compared with that of the unmodified carbon fiber-reinforced composites (1286 MPa). The grafting of carbon nanotubes can significantly increase the specific surface area and surface roughness of carbon fibers and enhance the mechanical "riveting" effect between carbon fibers and matrix resin; moreover, a large number of epoxy functional groups at the ends of CNTs can participate in the chemical reaction with carbon fibers and epoxy resin, which can introduce more chemical bonding at the interface of the two phases and enhance the interfacial adhesion. The interfacial adhesion is enhanced to the greatest extent. Therefore, when subjected to an applied load, more energy needs to be consumed to break the strong interface, and thus the bending strength can be improved.

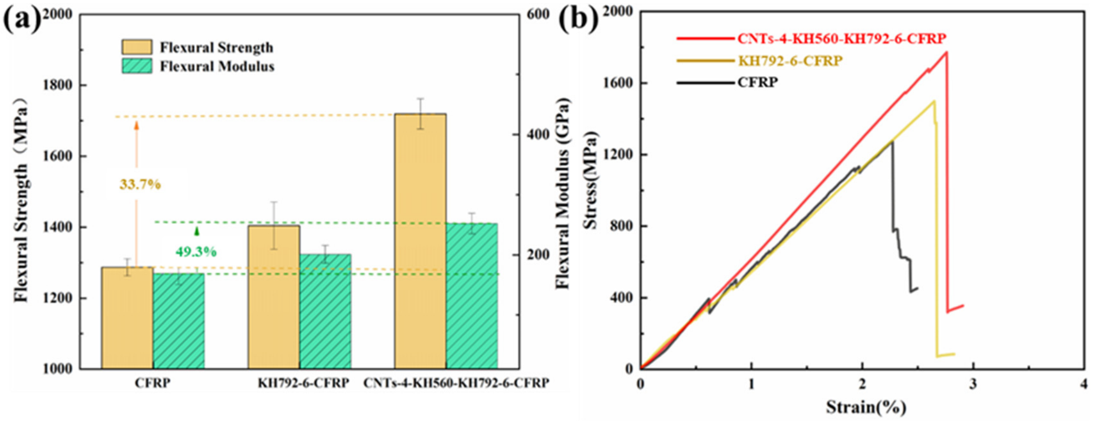


**Figure S3.** Experimental results of three-point bending of carbon fiber-reinforced composites: (a) flexural strength and flexural modulus, (b) stress–strain curve.

**Table S1.** Carbon fiber surface XPS split peak fitting results.

| Samples | Binding energy（eV）(%) | | | | | |
|---|---|---|---|---|---|---|
| | **288.9 -C=O** | **286.9 C-O** | **285.4 C-C** | **284.6 C=C** | **284.2 C-Si** | **286.1 C-N** |
| CF | 1.51 | 18.73 | 22.82 | 56.94 | 0 | 0 |
| KH792-6-CF | 0.63 | 17.82 | 15.56 | 35.84 | 15.30 | 14.85 |
| CNTs-4-KH560-KH792-6-CF | 1.88 | 19.47 | 19.08 | 37.49 | 16.84 | 5.24 |

**Table S2.** Mechanical properties of different samples.

| Samples | Tensile strength (MPa) | Performance enhancement | Tensile modulus (GPa) | Performance enhancement |
|---|---|---|---|---|
| CFRP | 519 | / | 12 | / |
| KH792-6-CFRP | 633 | 22.0% | 17 | 41.7% |
| CNTs-4-KH560-KH792-6-CFRP | 729 | 40.5% | 21 | 66.3% |
